# Calcium and Zinc Containing Bactericidal Glass Coatings for Biomedical Metallic Substrates

**DOI:** 10.3390/ijms150713030

**Published:** 2014-07-23

**Authors:** Leticia Esteban-Tejeda, Luis A. Díaz, Catuxa Prado, Belén Cabal, Ramón Torrecillas, José S. Moya

**Affiliations:** 1 Department of Biomaterials and Bioinspired Materials, Institute of Materials Science of Madrid (ICMM-CSIC), Cantoblanco, Madrid 28049, Spain; E-Mails: lesteban@icmm.csic.es (L.E.-T.); jsmoya@icmm.csic.es (J.S.M.); 2 Nanomaterials and Nanotechnology Research Center (CINN-CSIC)—Universidad de Oviedo (UO) —Principado de Asturias, El Entrego, San Martín del Rey Aurelio 33940, Spain; E-Mails: la.diaz@cinn.es (L.A.D.); c.prado@cinn.es (C.P.); r.torrecillas@cinn.es (R.T.); 3 Moscow State University of Technology (STANKIN), Vadkovskij per. 1, Moscow Oblast, Moscow 101472, Russia

**Keywords:** bactericidal glass, biocompatible glass, bactericidal glassy coating, ZnO-glass, soda-lime glass, biometal coating

## Abstract

The present work presents new bactericidal coatings, based on two families of non-toxic, antimicrobial glasses belonging to B_2_O_3_–SiO_2_–Na_2_O–ZnO and SiO_2_–Na_2_O–Al_2_O_3_–CaO–B_2_O_3_ systems. Free of cracking, single layer direct coatings on different biomedical metallic substrates (titanium alloy, Nb, Ta, and stainless steel) have been developed. Thermal expansion mismatch was adjusted by changing glass composition of the glass type, as well as the firing atmosphere (air or Ar) according to the biomedical metallic substrates. Formation of bubbles in some of the glassy coatings has been rationalized considering the reactions that take place at the different metal/coating interfaces. All the obtained coatings were proven to be strongly antibacterial *versus* *Escherichia coli* (>4 log).

## 1. Introduction

Biomaterials are used in different parts of the human body, as stents in blood vessels, heart valves, or, such as, replacement implants in elbows, hips, shoulders, teeth, and knees. Compatible mechanical properties with the specific application, biocompatibility, high corrosion resistance, and low wear, as well as a good osseointegration, are required for these materials [1,2,3,4]. Metals and alloys have been widely employed as biomedical materials in the field of medical implants because of their good mechanical properties. In this sense, the advantages of metals compared with ceramics and polymers are their high resistance to fracture, great strength, toughness, elasticity, and electrical conductivity. Titanium and its alloys and stainless steel 316L are the most common biomedical materials due to their biocompatibility and good mechanical and physical properties [5]. However, these materials have limitations. For instance, they are bioinert with body fluid. This could be a disadvantage because an early integration is needed [6]. In order to improve bone integration ability of metallic implants several studies, based on morphological, chemical, or biological surface modification, have been carried out [7]. Another critical problem of medical implants is related to infections, which have clinical and economic consequences. Most surgical infections are acquired during surgery through the skin or body of the patient and produce prolonged hospitalization, complex revision procedures, failure of the implant and secondary surgeries for its removal, increasing the economic and mortality rates. In the USA, 2,600,000 orthopedic implants are inserted annually, of which 112,000 became infected, which lead to an estimated average cost of combined medical and surgical treatment of $45,000 per case [8]. Because of this fact, nowadays there is an increase in the demand for new, long-lasting implants, it is estimated that by the end of 2030 the number of total hip replacements will rise by 174% (572,000 procedures) and total knee arthoplasties will grow by 673% (3.48 million of procedures) in US [9].

However, in spite of prevention methods, such as antibiotic prophylaxis, the use of gloves, drapes, masks, ultraviolet light during the surgery, and post-surgical wound care, the implants themselves are susceptible to develop infection on their surface. This is due to the formation of biofilm on the surface of the implant and the compromised immune response at the implant/tissue interface. In addition to this, bacteria in biofilm are protected from the immune defenses and are usually resistant to antibiotics, even high local concentrations of antibiotics cannot eradicate biofilms [10,11]. Therefore, to prevent bacterial adhesion on implant surface is considered today a critical objective.

There are several methods to prevent implants infections. Some of them involve a combination of metallic implants and local drug delivery that have two standout applications: cardiovascular and orthopeaedic and dental implants [10]. Drug delivery using metal is commonly embedding drugs into coatings applied to the metallic implant. The incorporation of the drug into the implant by covalent bonding [12,13], self-assembled monolayers [14], or embedding silver nanoparticles is also reported [15,16,17]. However, bactericidal inorganic coatings on metallic implants with biocompatibility have not been well-studied in the literature.

The present work presents new bactericidal coatings based on two families of non-toxic glasses belonging to B_2_O_3_–SiO_2_–Na_2_O–ZnO and SiO_2_–Na_2_O–Al_2_O_3_–CaO–B_2_O_3_ systems, which could avoid biofilm formation on the implant surface. ZnO containing glasses were previously studied as bactericide powders [18]. It is well reported that ZnO even in the form of nanoparticles exhibits a minimal effect on human and animal cells [19,20,21]. They are widely used as drug carriers, cosmetics ingredients, and medical filling materials due to their biocompatible and non-toxic character [22,23]. The other family of bactericidal proposed coating is based on soda-lime glasses with high content of CaO, which antimicrobial capability and biocompatibility has been previously proved [24,25]. Bioactivity of coating with one of these glasses (labeled as G3) on titanium alloy and on zirconium oxide substrates was studied in a previous study. The capability of G3 coating to form hydroxyapatite was pointed out. Precipitation of needle like hydroxyapatite crystals on the biocidal layer took place during the artificial saliva test [26].

Bioactive glasses have usually been combined with special antibacterial ions in order to achieve antibacterial properties. Most research in this field has dealt with the development of bactericidal Ag_2_O-doped bioactive glasses using different techniques including sol-gel [27] and ion exchange processes [28]. The advantage of using these glassy coatings *versus* other bioglasses reported in the literature [29,30] is that they deliver intrinsic and long-lasting bactericidal efficacy. They contain environmentally safe mineral elements essentials to humans, which release in a controlled manner avoiding possible toxic side effects. Even more, these glassy coatings may also be beneficial in preventing corrosion of implants in addition to antimicrobial and osseointegration properties. Titanium implants can corrode even in the absence of macroscopic wear mechanisms [31], therefore, a coating that can reduce this corrosion would be extremely beneficial.

The selected metallic substrates were the following: Ti6Al4V alloy, tantalum, niobium and stainless steel alloy 316L. They were selected based on their biocompatibility and the fact that they are widely used as metallic implants.

## 2. Results

### 2.1. Coating Characterization

**Figure 1:**
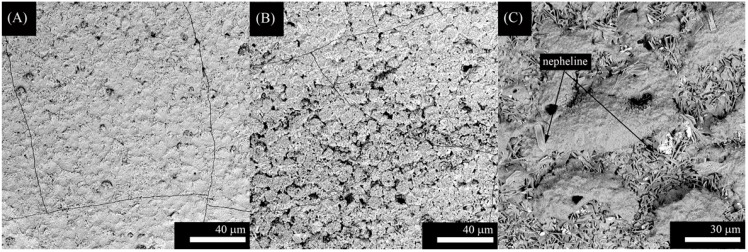
Scanning electronic micrographs of the top surface of the coatings with G3 on the following substrates: (**A**) Ti alloy in argon; (**B**) Ti alloy in air; and (**C**) stainless steel in air.

**Figure 2:**
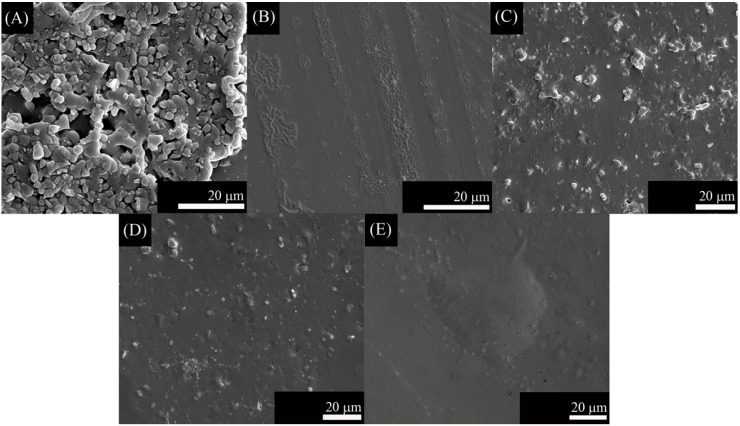
Scanning electronic micrographs of the top surface of ZnO15 coatings on: (**A**) Ti alloy in argon; (**B**) Ti alloy in air; (**C**) Ta in argon; (**D**) Nb in argon; and (**E**) stainless steel in air.

**Figure 3:**
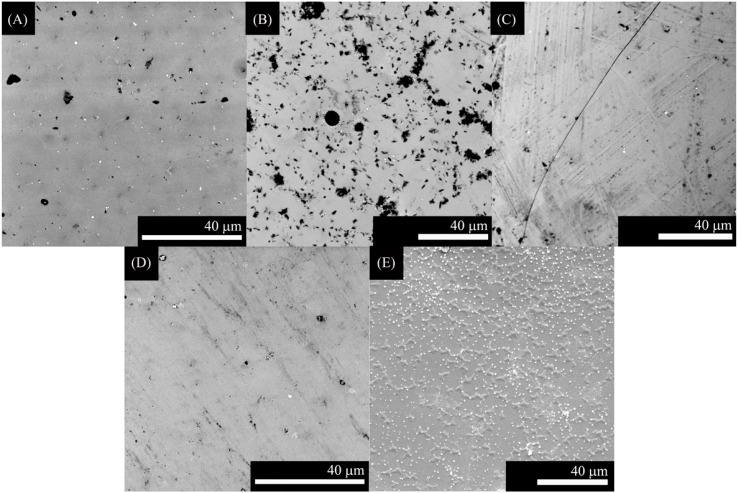
Scanning electronic micrographs of the top surface of ZnO35 coatings on the following substrates: (**A**) Ti alloy in argon; (**B**) Ti alloy in air; (**C**) Ta in argon; (**D**) Nb in argon; and (**E**) stainless steel in air.

**Figure 4:**
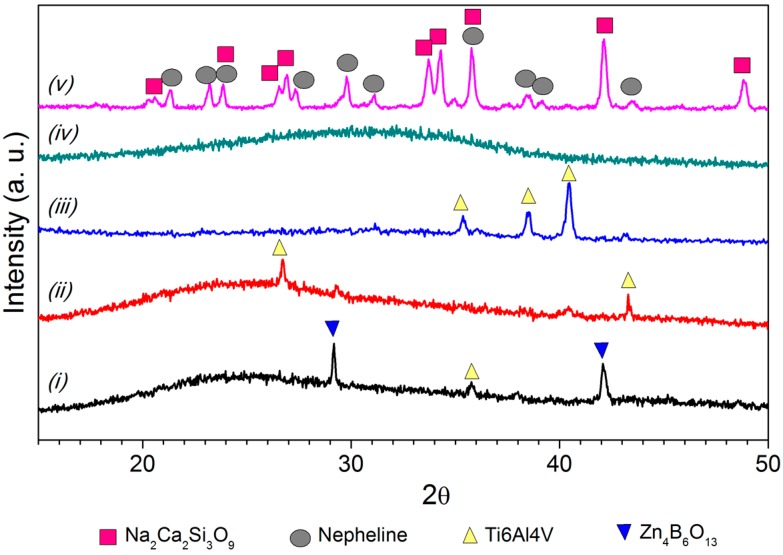
XRD patterns of the top surface of the coatings on Ti6Al4V substrate for: (i) ZnO15 heated in air; (ii) ZnO15 heated in argon; (iii) ZnO35 heated in air; (iv) ZnO35 heated in argon; and (v) G3 heated in air.

SEM micrographs from the top surface of G3, ZnO15 and ZnO35 coatings are shown in Figure 1, Figure 2 and Figure 3, respectively. In the case of G3 on Ti alloy (Figure 1A,B), cracking is observed due to the thermal expansion coefficients mismatch (Table 1, Δα = 4.5 × 10^−6^·K^−1^). To avoid this fact, it was necessary to precoat the plate with a window glass type having an intermediated α values, as it was reported in a previous work [30]. Due to the high thermal expansion coefficient of the stainless steel alloy (Table 1), coating with G3 glass is the most suitable for this substrate (Figure 1C). In this particular case, after thermal treatment at 750 °C the original G3 glass devitrifies given 2 crystalline phases: nepheline (Figure 1C and Figure 4) and combeite (Figure 4). Combeite crystals were not observed by SEM due to their small size.

**Table 1:** Thermal expansion coefficients (α).

α (10^−6^·K^−1^)						
ZnO15	ZnO35	G3	Ti Alloy	Ta	Nb	Stainless Steel 316L
7.7	10.7	14.2	9.7	6.3	7.3	16.0

**Figure 5:**
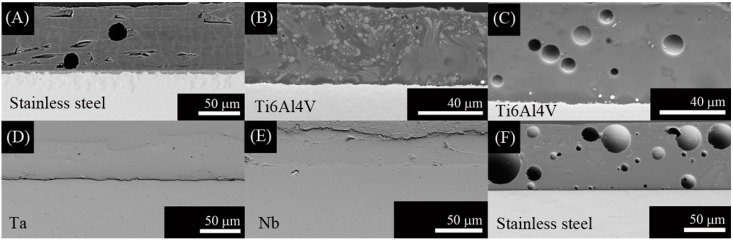
Scanning electronic micrographs of the cross section of the coating with: (**A**) G3 on stainless steel and with ZnO15 on the following substrates; (**B**) Ti alloy in argon; (**C**) Ti alloy in air; (**D**) Ta in argon; (**E**) Nb in argon; and (**F**) stainless steel in air.

In the present work, we have tried to obtain a single layer direct coating on the metal substrate. In this regard, we adjusted the thermal expansion mismatch (Table 1) by changing the bactericidal ZnO containing glass composition according to the substrates used (Ti alloy, Ta, or Nb). Coatings using ZnO15 show well-dispersed particles that resulted in no cracking (Figure 2). Similarly, ZnO35 coatings were absent of cracks, except for ZnO35 coating on Ta (Figure 3C), in which a slight cracking can be observed due to the thermal expansion coefficient mismatch (Table 1, Δα = 4.4 × 10^−6^·K^−1^), as occurred in the case of G3 on Ti alloy (Figure 1A,B). However, the study of the cross section shows that this coating has a good adherence to the Ta substrate (Figure 5D).

**Figure 6:**
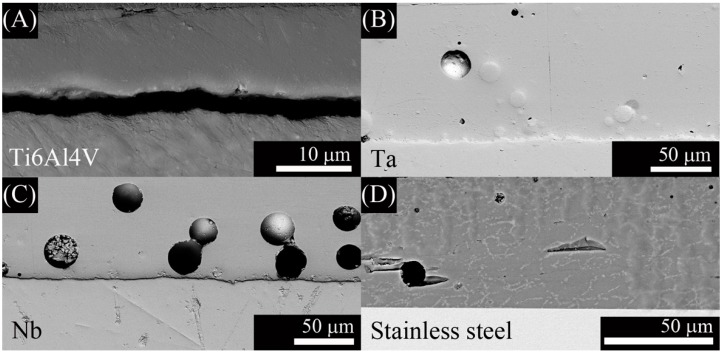
Scanning electronic micrographs of the cross section of the coating with ZnO35 on the following substrates: (**A**) Ti alloy in air; (**B**) Ta in argon; (**C**) Nb in argon; and (**D**) stainless steel in air.

Cross section of the coatings to study the adherence and the reactions at the interface are shown in Figure 5 and Figure 6. Cross section of G3 on Ti alloy is reported in a previous work [26].

**Figure 7:**
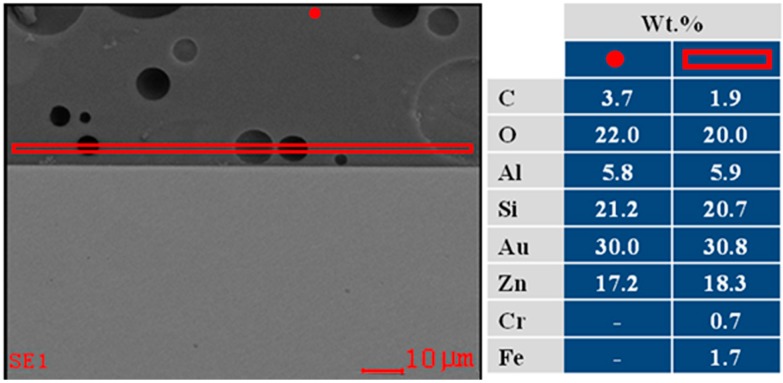
FE**-** SEM micrograph of the cross section of ZnO15 coating on stainless steel alloy in air (**on the left**); and energy dispersive spectrometry (EDS) analysis of the selected area (**on the right**). The areas selected were one close to the interface (**red rectangle**) and the other far away from it (**red point**).

Energy dispersive spectrometry (EDS) analysis on the top surface and at the interface of the ZnO15 coating on stainless steel alloy in air is shown in Figure 7.

All XRD patterns from the top surface were similar because the reactions take place at the interface, not at the bulk. As an example, only the coatings on Ti6Al4V substrate are shown in Figure 4. A zinc borate (Zn_4_B_6_O_13_ ICDD: 019-1261) was observed in the case of ZnO containing glasses. In the case of the G3 glass, two crystalline phases (combeite (Na_2_Ca_2_Si_3_O_9_ FIZ 075-1686) and nepheline (Na_6.65_ Al_6.24_ Si_9.76_ O_32_ FIZ 083-2372)) were identified [25,26].

### 2.2. Bactericidal Activity

**Figure 8:**
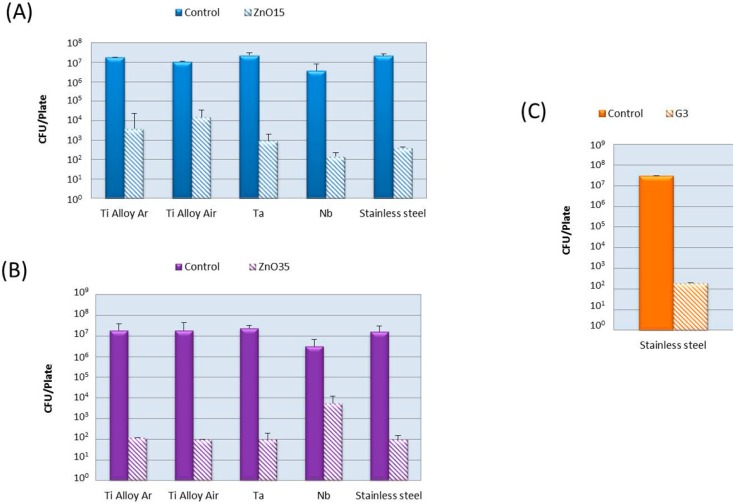
Antibacterial activity against *E. coli* of the coatings with: (**A**) ZnO15; (**B**) ZnO35 and (**C**) G3. Bars represent media (SD) from three replicates.

The antibacterial activity was evaluated against *E. coli* and the results are presented in Figure 8. The antimicrobial effectiveness was studied based on the logarithm reduction in viable counts of the tests bacteria. It was calculated by subtracting the log_10_ colony counts in the control sample (no biocide added) from those present in the problem samples. It can be seen that all coatings achieve a logarithm reduction >4, that means a safe disinfection. ZnO35 coatings have the highest antibacterial activity attributed to their higher ZnO content.

## 3. Discussion

The absence of cracking and detachment at the interface were signals of mechanical stable coatings. However, further quantitative characterization to determine the adhesion is required. This will be the aim of a future work. More bubbles were observed in the ZnO15 coatings heat treated in air (Figure 5C,F). This is a consequence of the chemical reactions that take place at the interface. First, gas diffuses through porous coating and a thin oxide layer is formed on the metal surface. At higher temperatures, glass-softening occurs dissolving the already formed oxide layer and starts to react with the substrate. The P_(O2)_ at the furnace is relevant to the extension of the surface oxidation of the metal substrates. In air, the oxidation is much higher so the glass reacts more with the oxide layer forming more silicides, which are undesirable because they are usually brittle and produce dewetting and result in bubble formation due to the liberation of oxygen and sodium gas during the reactions [32,33]. Reactions for Me = (Ti alloy, Ta, Nb) are shown below [32,33]. These metals are considered highly reactive due to their ΔG° for the formation of the corresponding oxides (Table 2) is highly negative, indicating that the oxides are highly stable.

Preoxidation of the metal (∆ *G* ° < 0, Table 2):

Me + ½O*_2_* ⟷ MeO(int) (1)

Glass dissolves the oxide layer:

MeO(int) ⟷ MeO(glass)
(2)

Oxide redox reactions:

Me + Na*_2_*O(glass) ⟷ MeO(int) + 2Na ↑ (3)

Silicides formation:

5Me + 3SiO_2_(glass) ⟷ Me_5_Si_3_ + 3O_2_ (4)

8Me + 3SiO_2_(glass) ⟷ Me_5_Si_3_ + 3MeO_2_ (5)

∆G°_1026.8 °C_ for reactions 1 and 3 are given in Table 2. ∆G° is positive for equation 3. Conditions under which ∆G becomes negative are determined by its equilibrium constant shown in equation 6. Reaction is favored with a low p (Na), a low activity of MeO (int) and a high activity of Na_2_O (glass). The pressure of the formed Na vapor has to exceed ambient pressure to nucleate bubbles at the interface and escape; therefore this reaction is favored in vacuum or low-pressure atmosphere.

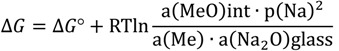
(6)


Another type of redox reaction is the reduction of Si^4+^ in the glass and the oxidation of the metal substrate to form a silicide layer at the interface, if the metal has enough oxidation potential as it is the case of Ti, Ta, and Nb (Reactions 4 and 5).

Stainless steel alloy is a different case because it contains Fe that is a reactive metal, but less than Ti, Ta, and Nb, as its lower Δ *G* ° indicates for the oxide formation (Table 2). Stainless steel also contains Cr that is a highly reactive metal, similar to Ti. Reactions that take place in this particular case are: Preoxidation of the metal (∆ *G* ° < 0, Table 2):

Fe + ½O*_2_* ⟷ MeO(int) (7)

Glass dissolves the oxide layer:

FeO(int) ⟷ FeO(glass)
(8)

Oxide redox reactions with the corresponding ΔG equation:

Fe + Na_2_O(glass) ⟷ FeO(int) + 2Na ↑ (9)

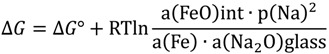
(10)

2FeO(glass) + Na_2_O(glass) ⟷ Fe_2_O_3_(glass) + 2Na ↑ (11)

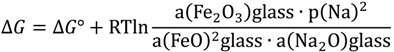
(12)

Cr + 3/2 Na_2_O(glass) ⟷ 1/2 Cr_2_O_3_ + 3Na ↑ (13)

2Cr + SiO_2_(glass) ⟷ 2CrO + Si (14)

(1 + 2x)Cr + xSiO_2_(glass) ⟷ CrSi_x_ + 2xCrO(glass) (15)

**Table 2:** Δ*G*°_1026.8 °C_ for reactions 1, 3, 7, and 9 [32,33].

Metal	Δ *G*°_1026.8 °C_ for Reactions 1 and 7 (kcal/mol)	Δ *G*°_1026.8 °C_ for Reactions 3 and 9 (kcal/mol)
Fe	−89.9	65.5
Ta	−141.6	198.3
Nb/Nb^2+^	−144.6	38.1
Nb/Nb^5+^	−128.0	232.4
Ti/Ti^2+^	−200.2	10.4
Ti/Ti^3+^	−185.5	53.1
Ti/Ti^4+^	−168.2	52.8

In accordance with this thermodynamic analysis, bubbles were observed in ZnO15 coatings heated in air on Ti6Al4V alloy (Figure 5C) and on stained steel (Figure 5F). Based on this, it is possible to conclude that the best coating fabricated with ZnO15 was in argon. In the case of ZnO35, bubbles were also observed in coatings heated in argon on Ta (Figure 6B) and on Nb (Figure 6C), probably due to the lower viscosity of the ZnO35 glass at 630 °C that hampers the liberation of the gas formed, due to interfacial reactions (3 and 4) which take place during the heat treatment. In the particular case of the Ti6Al4V substrate (Figure 6A), bubbles were not observed because there is no contact between the coating and the substrate, which means that an excessive reaction and formation of silicides take place. Silicides are undesirable because they are brittle and have a higher thermal expansion coefficient than Ti6Al4V alloy, so they produce a bad adherence to the substrate.

EDS analysis was carried out along the interface and close to the surface of the coatings in order to illustrate the reactions at the interface. FE-SEM-EDS of ZnO15 coating on stainless steel alloy in air (Figure 7) confirms the presence of iron near to the interface (Figure 7B) and its lack on the top surface (Figure 7A). This means that iron from the stainless steel alloy reacts with the glass forming FeO, which has been incorporated into the glassy matrix of the coating. 

Regarding the bactericidal activity, all the coatings were strongly antibacterial against *E.* *coli*. The antimicrobial activity of glass labeled as G3, arises from the capability to release calcium ions at the glass-particle interface, which leads to membrane depolarization and the subsequent death of the cell, as is reported in a previous work [24]. In the case of ZnO containing glasses, there are several possible mechanisms for the antibacterial action of zinc oxide. The generation of radical oxygen species (ROS), such as hydrogen peroxide (H_2_O_2_), superoxide anion (O_2_^−^) and hydroxyl radicals (OH^−^), are considered as an effective means for the inhibition of bacterial growth [34]. Another possible mechanism for ZnO antibacterial activity is the release of high zinc ion concentrations, which can damage cell membrane and interact with intracellular contents [19]. In a previous work [18], Zn^2+^ released from glasses powder (<30 mm) was found to be ~400 ppm. In the case of the coatings, Zn^2+^ release must be significantly lower due to their lower specific surface area in comparison with glass powder.

It is critical to find a correct balance between bactericidal effects and biocompatibility properties. In the case of the G3 coatings, *in vitro* biocompatibility tests by using mesenchymal stem cells derived from human bone have been carried out in a previous work [25]. The results obtained in this investigation demonstrate that this glass-ceramic has an excellent biocompatibility. On the other hand, in the case of Zn based glasses, *in vitro* biocompatibility assays with human mesenchymal stem cells, fibroblasts and immune cells (monocytes and lymphocytes) are currently ongoing. Although preliminary results show no evidence of cytotoxicity, further tests are required.

## 4. Experimental Section

### 4.1. Materials

Three antimicrobial glasses were used as a precursor of the bactericidal coatings: (i) a soda-lime glass from the SiO_2_–Na_2_O–Al_2_O_3_–CaO–B_2_O_3_ system labeled as G3 [24]; and (ii) two glasses belonging to the B_2_O_3_–SiO_2_–Na_2_O–ZnO system with the following ZnO content (wt %): 15 and 35, labeled as ZnO15 and ZnO35, respectively [18]. They were prepared by melting appropriate mixtures of reagent grade SiO_2_, α-Al_2_O_3_, H_3_BO_3_, Na_2_CO_3_, CaCO_3_, ZnO. The starting materials were weighed, mixed and melted in a Pt crucible for 1 h at 850 °C to favor decarbonation of samples, and subsequently for 1 h at 1400 °C (G3) and at 1250 °C (ZnO15, ZnO35). The melts were then quenched in water and grounded by ball milling to fine particles, and sieved to obtain particle size <30 μm. Thermal properties of the glasses were investigated by differential thermal analysis (DTA-Tg) in previous works. No exothermic peaks were observed in the case of ZnO based glasses. Whereas G3 glass, exhibits two well-defined crystallization exotherms at around 645 and 700 °C, attributed to the crystallization of combeite and nepheline crystals [25]. The composition and main thermal properties of the glasses [hemisphere temperature (T_h_), transition temperature (T_g_) and softening temperature (T_s_)] are listed in Table 3. The following metals plates (25 × 25 × 1 mm^3^) provided for Goodfellow were used as substrate: Ti6Al4V annealed, Niobium 99.9% purity, Tantalum 99.9% purity and stainless steel AISI 316L (Fe/Cr18/Ni10/Mo3) annealed. Thermal expansion coefficients are summarized in Table 1.

**Table 3:** Chemical composition (mol %) and thermal properties.

Glass	SiO_2_	B_2_O_3_	Na_2_O	CaO	Al_2_O_3_	K_2_O	ZnO	T_h_	T_g_	T_s_
ZnO15	29.4	45.0	8.2	–	4.6	–	12.8	715	487	513
ZnO35	23.1	35.3	6.4	–	3.6	–	31.6	666	475	510
G3	43.0	7.8	19.4	22.0	7.4	0.4	–	934	507	535

### 4.2. Preparation of the Coatings

Coatings were prepared following two different procedures: (i) one was by decantation. Briefly, a suspension of glass powder (<30 μm) in water was poured onto the plates placed in a beaker. The suspension of particles was dispersed using ultrasounds in order to achieve a uniform distribution of particles on the coating surface. Afterwards, the liquid was removed with a pipette and the beaker was placed in an oven at 100 °C overnight to dry the green-coatings completely; (ii) The other one method was by screen-printing technology. A polymer ink, based on a mixture of epoxy and glass powder, was prepared for the glass layer screen-printing. This layer was deposited on the corresponding substrate surface.

The green-coatings prepared as indicated were fired in air or argon atmosphere (temperature ramp 10 °C/min) at 725 °C for ZnO15, 630 °C for ZnO35, and 750 °C for G3, according with their glass hemisphere and transition temperatures (Table 3). No significance effect on viscosity of these glasses is expected at the studied temperatures according to Ellingham diagrams. Ta and Nb substrates were heat treated in argon to avoid their oxidation. Ti alloy was studied in both atmospheres, argon and air, and stainless steel 316L in air. It is important to point out that in the selected temperature range (650–750 °C) and atmospheres the mechanical properties of the titanium alloy substrate are not significantly affected [15].

### 4.3. Coatings Characterization

All glasses were isostatically pressed at ~200 MPa into bars of ø 4 mm and sintered in air for 1 h at 725, 630, and 750 °C for ZnO15, ZnO35 and G3, respectively, in order to determine their thermal expansion coefficients (Table 1). They were measured in a BÄHR THERMOANALYSE model DIL 802 (TA Instruments, Hüllhorst, Germany) in air. XRD analyses were carried out using a Bruker D8 with CuKα radiation working at 40 kV and 30 mA in a step-scanning mode with a step width of 0.0288 and a step time of 2.5 s. Thermal properties of the glasses (transition temperature (T_g_) and softening temperature (T_s_)) were investigated by differential thermal analysis (DTA-Tg, Stanton Instrument LTD, London, UK) (Stanton Mod. STA 781). A side view hot stage microscope (HSM) EM 201, with image analysis system and electrical furnace, 1750/15 Leica, was used to determine hemisphere temperature (T_h_). All microstructures were studied by using Field Emission Scanning Electron Microscopy (FE-SEM, FEI, Hillsboro, OR, USA) (FEI: Quanta FEG650) with an associated energy dispersive spectroscopy analysis (EDS, FEI, Hillsboro, OR, USA) (EDAX-AMETEK). Samples were coated with an Au nanometric conductive layer for FE-SEM observations.

### 4.4. Bactericidal Test

Measurement of the antibacterial activity was carried out following ISO 22196 standard method (Measurement of antibacterial activity on plastics and other non-porous surfaces [35]). Briefly, the coated face of the plate was inoculated with 100 µL of melted soft agar (LB with 0.6% agar) containing ~10^7^ bacteria per mL. Then, the microbial inoculum was covered with a thin and sterile film. The covering allows the inoculum to spread well, prevents it from drying, and ensures good contact with the bactericidal surface. Inoculated and covered surfaces were incubated in a humid environment at 37 °C for 40 h. After incubation, the agar was removed, cut into small pieces and shaken for 6 h in PBS to allow bacteria to diffuse out of the agar. The number of microorganisms was determined by serial dilution plating.

Non-coated plates of titanium alloy, tantalum, niobium and stainless steel alloy 316L were tested as negative controls. Assays were carried out by triplicate. The microorganism studied was *Escherichia coli DH10B*. *E. coli* is one of the most frequent pathogens implicated in the etiology of biomaterials-associated infections [36].

## 5. Conclusions

Following conclusions can be drawn from the results obtained in this investigation: 

Mechanically stable bactericidal direct glassy coatings of ZnO15 were fabricated at 725 °C on:

- Ti6Al4V and stainless steel 316L in air;
- Ti6Al4V, Ta, and Nb in argon atmosphere.

Mechanically stable bactericidal direct glassy coatings of ZnO35 were fabricated at 630 °C on:

- Stainless steel 316L in air;
- Ta, and Nb in argon atmosphere.

Mechanically stable bactericidal direct glassy coating of G3 was fabricated at 750 °C on stainless steel 316L in air.

All the obtained coatings were proven to be strongly antibacterial *versus* *E.coli* (>4 log).

## References

[1] Geetha M., Singh A.K., Asokamani R., Gogia A.K. (2009). Ti based biomaterials, the ultimate choice for orthopaedic implants—A review. Prog. Mater. Sci..

[2] Park J.B., Bronzino J.D. (2003). Biomaterials: Principles and Applications.

[3] Ramakrishna S., Mayer J., Wintermantel E., Leong K.W. (2001). Biomedical applications of polymer-composite materials: A review. Compos. Sci. Technol..

[4] Wise D.L. (2000). Biomaterials Engineering and Devices.

[5] Long M., Rack H.J. (1998). Titanium alloys in total joint replacement: A materials science perspective. Biomaterials.

[6] Ha J.Y., Tsutsumi Y., Doi H., Nomura N., Kim K.H., Hanawa T. (2011). Enhancement of calcium phosphate formation on zirconium by micro-arc oxidation and chemical treatments. Surf. Coat. Technol..

[7] Spriano S., Ferraris S., Rimondini L. (2012). Metallic surfaces for osteointegration. Surface Tailoring of Inorganic Materials for Biomedical Applications.

[8] Rabih O., Darouiche M.D. (2004). Treatment of infections associated with surgical implants. N. Engl. J. Med..

[9] Kurtz S., Ong K., Lau E., Mowat F., Halpern M. (2007). Projections of primary and revision hip and knee arthroplasty in the United States from 2005 to 2030. J. Bone Jt. Surg..

[10] Lyndon J., Boyd B., Birbilis N. (2014). Metallic implant drug/device combinations for controlled drug release in orthopaedic applications. J. Control. Release.

[11] Matl F.D., Obermeier A., Repmann S., Friess W., Stemberger A., Kuehn K.D. (2008). New anti-infective coatings of medical implants. Antimicrob. Agents Chemother..

[12] Antoci V., King S.B., Jose B., Parvizi J., Zeiger A.R., Wickstrom E., Freeman T.A., Composto R.J., Ducheyne P., Shapiro I.M. (2007). Vancomycin covalently bonded to titanium alloy prevents bacterial colonization. J. Orthop. Res..

[13] Jose B., Antoci V., Zeiger A.R., Wickstrom E., Hickok N.J. (2005). Vancomycin covalently bonded to titanium beads kills *Staphylococcus aureus*. Chem. Biol..

[14] Mani G., Johnson D.M., Marton D., Feldman M.D., Patel D., Ayon A.A., Agrawal C.M. (2008). Drug delivery from gold and titanium surfaces using self-assembled monolayers. Biomaterials.

[15] Esteban-Tejeda L., Cabal B., Malpartida F., López-Piriz R., Torrecillas R., Saiz E., Tomsia A.P., Moya J.S. (2012). Soda-Lime glass coating containing silver nanoparticles on Ti-6Al-4V alloy. J. Eur. Ceram. Soc..

[16] López Píriz R., Solá Linares E., Granizo J., Díaz Güemes I., Enciso S., Bartolomé J., Cabal B., Esteban Tejeda L., Torrecillas R., Moya J. (2012). Radiologic evaluation of bone loss at implants with biocide coated titanium abutments: A study in the dog. PLoS One.

[17] Zheng Y., Li J., Liu X., Sun J. (2012). Antimicrobial and osteogenic effect of Ag-implanted titanium with a nanostructured surface. Int. J. Nanomed..

[18] Esteban-Tejeda L., Prado C., Cabal B., Sanz J., Torrecillas R., Moya J.S. (2014). Antibacterial and antifungal activity of ZnO non-toxic and environmentally friendly glasses. Environ. Sci. Technol..

[19] Brayner R., Ferrari Iliou R., Brivois N., Djediat S., Benedetti M., Fiévet F. (2006). Toxicological impact studies based on *Escherichia coli* bacteria in ultrafine ZnO nanoparticles colloidal medium. Nano Lett..

[20] Huh A., Kwon Y. (2011). “Nanoantibiotics”: A new paradigm for treating infectious diseases using nanomaterials in the antibiotics resistant era. J. Control. Release.

[21] Reddy K.M., Feris K., Bell J., Wingett D., Hanley C., Punnoose A. (2007). Selective toxicity of zinc oxide nanoparticles to prokaryotic and eukaryotic systems. Appl. Phys. Lett..

[22] Jiang P., Zhou J.J., Fang H.F., Wang C.Y., Wang Z.L., Xie S.S. (2007). Hierarchical shelled ZnO structures made of bunched nanowire arrays. Adv. Funct. Mater..

[23] Zhou J., Xu N.S., Wang Z.L. (2006). Dissolving behavior and stability of ZnO wires in biofluids: A study on biodegradability and biocompatibility of ZnO nanostructures. Adv. Mater..

[24] Moya J.S., Esteban-Tejeda L., Pecharromán C., Mello-Castanho S.R.H., da Silva A.C., Malpartida F. (2011). Glass-powders with a high content of calcium oxide: A step toward a “Green” universal biocide. Adv. Biomater..

[25] Cabal B., Alou L., Cafini F., Couceiro R., Sevillano D., Esteban-Tejeda L., Guitián F., Torrecillas R., Moya J.S. (2014). A new biocompatible and antibacterial phosphate free glass-ceramic for medical applications. Sci. Rep..

[26] Esteban Tejeda L., Díaz L.A., Cabal B., Prado C., López Piriz R., Torrecillas R., Moya J.S. (2013). Biocide glass–ceramic coating on titanium alloy and zirconium oxide for dental applications. Mater. Lett..

[27] Bellantone M., William H.D., Hench L.L. (2002). Broad-spectrum bactericidal activity of Ag2O-doped bioactive glass. Antimicrob. Agent Chemother..

[28] Verné E., Nunzio S.D., Bosetti M., Appendino P., Vitale Brovarone C., Maina G., Cannasb M. (2005). Surface characterization of silver-doped bioactive glass. Biomaterials.

[29] Aina V., Malavasi G., Fiorio Pla A., Munaron L., Morterra C. (2009). Zinc-containing bioactive glasses: Surface reactivity and behaviour towards endothelial cells. Acta Biomater..

[30] Verne E., Verné E., Ferraris S., Miola M., Fucale G., Maina G., Robotti P., Bianchi G., Martinasso G., Canuto R.A. (2008). Synthesis and characterisation of bioactive and antibacterial glass-ceramic Part 2—Plasma spray coatings on metallic substrates. Adv. Appl. Ceram..

[31] Addison O., Davenport A.J., Newport R.J., Kalra S., Monir M., Mosselmans J.F.W., Proops D., Martin R.A. (2012). Do “passive” medical titanium surfaces deteriorate in service in the absence of wear?. J. R. Soc. Interface.

[32] Pazo A., Saiz E., Tomsia A.P. (1998). Silicate glass coatings on Ti-based implants. Acta Mater..

[33] Tomsia A.P., Pask J.A. (1986). Chemical reactions and adherence at glass/metal interfaces: An analysis. Dent. Mater..

[34] Yousef J.M., Daniel E.N. (2012). *In vitro* antibacterial activity and minimum inhibitory concentration of zinc oxide and nano-particle zinc oxide against pathogenic strains. J. Health Sci..

[35] ISO 22196 Standard Method Measurement of Antibacterial Activity on Plastics and Other Non-Porous Surfaces. http://www.iso.org/.

[36] Moriarty F.Z., Sebastian A.J., Busscher H.J. (2013). Biomaterials Associated Infection.

